# Supplementary material for: Cinnamomum zeylanicum Blume Essential Oil Inhibits Metastatic Melanoma Cell Proliferation by Triggering an Incomplete Tumour Cell Stress Response
Source: Int J Mol Sci. 2023 Mar 16;24(6):5698. doi: 10.3390/ijms24065698 (PMC10058067; doi:10.3390/ijms24065698)
Supplement: Supplementary file 1 [file ijms-24-05698-s001.zip › ijms-2113722-supplementary.pdf]

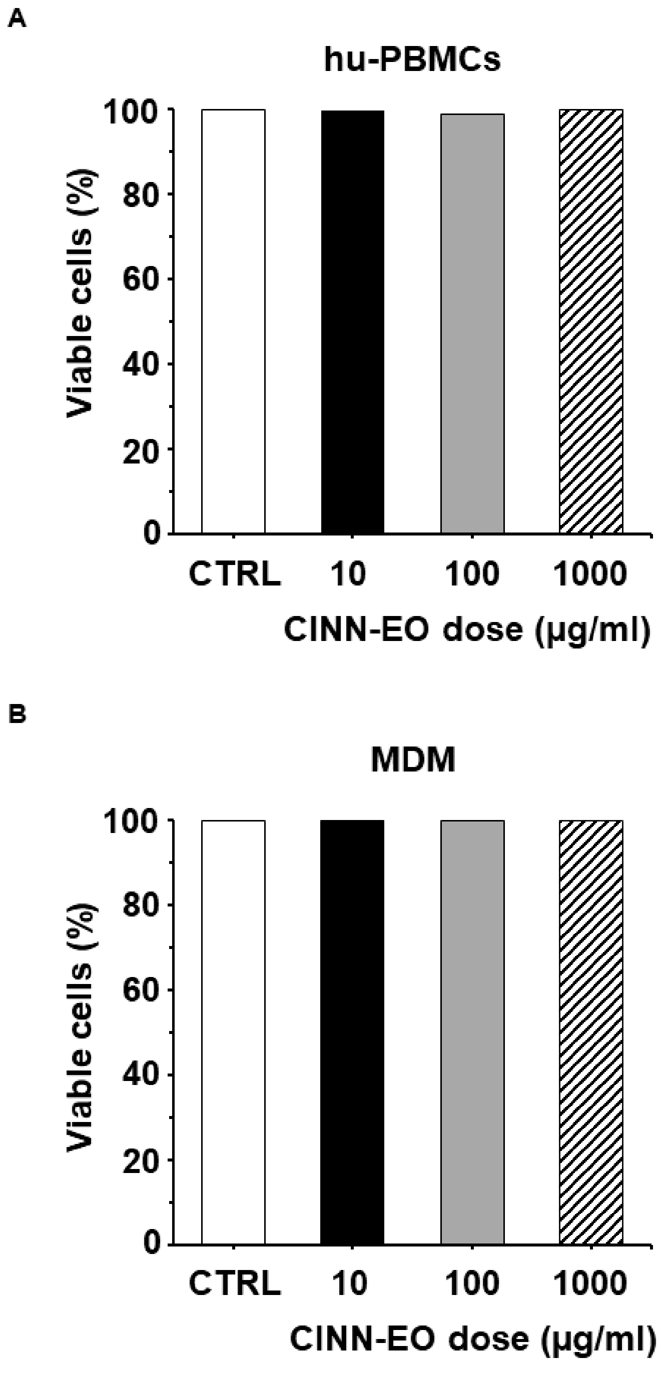

Figure S1. Dose-response effect of CINN-EO on healthy human PBMCs (A) and MDMs (B), obtained from healthy donors. CINN-EO was administered at the indicated concentrations for 24 h. The viable cells after each treatment is reported as percent of untreated control (CTRL).

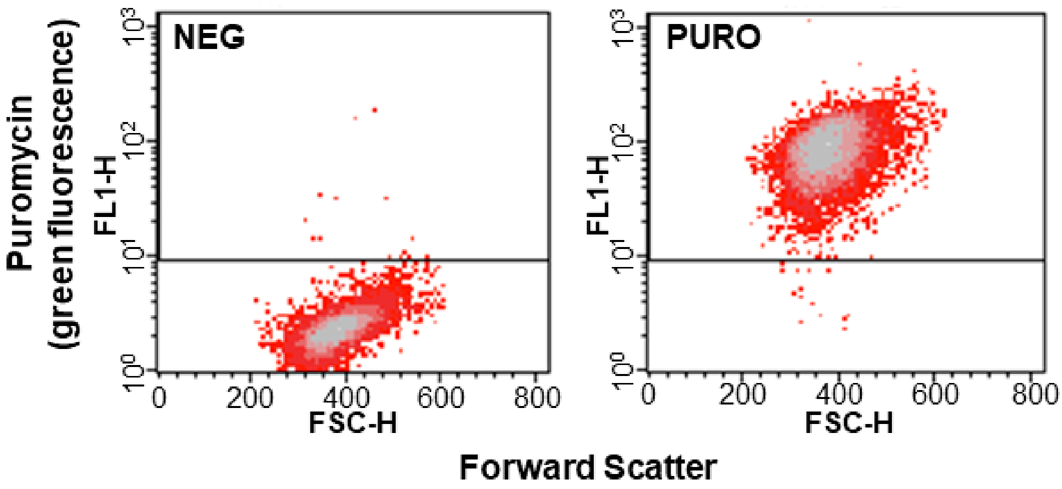

Figure S2. Representative cytograms of puromycin FACS expression. M14 cells were exposed to 1 µM puromycin for 4 h and then analysed by FACS for immunofluorescence. NEG, negative control without primary antibody; PURO, puromycin.

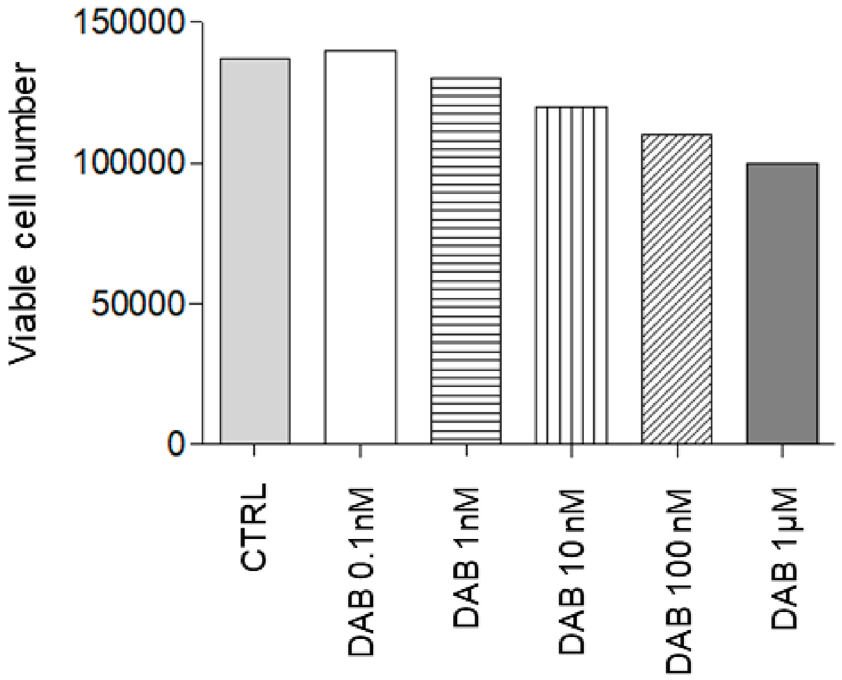

Figure S3. Dose-response effect of Dabrafenib (DAB) on M14 cells. DAB was administered at the indicated concentrations for 24 h. The number of viable cells after each treatment is reported.

Table S1. Compositions of the cinnamon essential oil samples.

#[a]	RI Lit.[b]	RI Exp.[c]	Class/Compound	EO1	EO2	EO3	EO4	EO5	EO6
				%					
			**Monoterpene hydrocarbons (16)**	**7.48**	**8.36**	**0.23**	**11.07**	**9.95**	**3.26**
3	927	926	α-Thujene	0.17	0.20			0.25	0.07
4	939	933	α-Pinene	1.16	1.34	0.08	1.48	1.07	0.74
5	954	948	Camphene	0.44	0.49	0.05	0.59	0.41	0.26
7	975	972	Sabinene	0.01	0.02	0.03	0.03	0.08	
8	979	975	β-Pinene	0.43	0.49		0.38	0.36	0.26
9	991	987	Myrcene	0.09	0.12		0.15	0.10	
10	1003	1000	α-Phellandrene	1.26	1.48		1.30	0.91	0.24
11	1009	1015	δ-3-carene	0.11	0.12				
12	1017	1014	α-Terpinene	0.29	0.28		1.07	0.93	0.06
13	1025	1022	*p*-Cymene	1.85	1.88	0.02	1.26	1.18	1.06
14	1029	1029	Limonene			0.05			0.56
15	1030	1028	β-Phellandrene	1.53	1.77		4.34	4.26	
17	1037	1036	β-*Z*-Ocimene	0.01	0.04		0.06	0.06	
19	1050	1047	β-*E*-Ocimene	0.03	0.03		0.04	0.04	
20	1060	1058	γ-Terpinene				0.19	0.18	
22	1089	1086	Terpinolene	0.10	0.12		0.16	0.13	
			**Oxygenated monoterpenes (14)**	**4.95**	**5.22**	**0.13**	**3.62**	**6.68**	**3.10**
16	1031	1031	1,8-Cineole	0.15	0.20		0.08	1.05	0.12
23	1097	1097	Linalool	4.18	4.38		1.29	3.82	2.64
26	1121	1121	dehydro-Sabine Ketone				0.15		
27	1122	1122	*cis-p*-Menth-2-en-1-ol				0.11	0.13	
28	1141	1141	*trans-p*-Menth-2-en-1-ol					0.09	
29	1146	1157	Camphor	0.01	0.03				
31	1169	1168	Borneol	0.04	0.03	0.06	0.06	0.05	
32	1177	1177	Terpinen-4-ol	0.19	0.17		1.27	0.53	0.07
33	1183	1191	*p*-Cymen-8-ol	0.03	0.04				
34	1189	1190	α-Terpineol	0.36	0.37		0.48	0.83	0.26
36	1221	1207	*cis*-Sabinene hydrate				0.06	0.05	
44	1291	1288	*trans*-Sabinyl acetate			0.07			
47	1290	1294	Thymol				0.09	0.08	
48	1299	1302	Carvacrol				0.02	0.05	
			**Sesquiterpenes (29)**	**4.87**	**4.80**	**0.75**	**3.36**	**3.60**	**5.74**
53	1377	1379	α-Copaene			0.15	0.11	0.13	0.61
55	1387	1413	β-Cubebene	0.05					
57	1408	1426	*iso*-Caryophyllene	0.01	0.03				
58	1419	1425	β-Caryophyllene	3.99	3.98	0.07	2.27	2.00	2.25
60	1460	1455	*allo*-Aromadendrene			0.03			
61	1455	1461	α-Humulene	0.65	0.65		0.42	0.34	0.39
62	1481	1477	γ-Muurolene			0.05			
63	1481	1486	*ar*-Curcumene	0.02		0.02	0.03		

64	1493	1505	Guajene	0.03	0.04				
65	1498	1490	α-Selinene					0.04	
66	1500	1501	α-Muurolene			0.02	0.04	0.06	
67	1506	1502	β-Bisabolene			0.03		0.07	
68	1496	1515	Viridiflorene	0.01					
69	1516	1518	β-Curcumene			0.04			
71	1523	1527	δ-Cadinene	0.12	0.10	0.06		0.08	
73	1546	1550	α-Calacorene			0.02			
74	1572	1562	Caryophyllene alcohol				0.02	0.25	
75	1563	1570	*E*-Nerolidol			0.06			
76	1578	1581	Spathulenol			0.09	0.08	0.06	0.09
77	1583	1594	Caryophyllene oxide				0.32	0.30	1.49
78	1585	1595	Globulol			0.08			
80	1608	1613	Humulene epoxide						0.24
81	1629	1615	1-*epi*-Cubenol					0.04	
82	1640	1648	*epi*-α-Cadinol					0.05	
83	1646	1652	α-Muurolol					0.03	
84	1660	1662	Intermedeol					0.06	
85	1670	1670	14-hydroxy-9-*epi*-*E*-Caryophyllene				0.09	0.05	0.17
86	1682	1677	Occidentalol acetate					0.04	0.52
87	1686	1690	α-Bisabolene			0.02			
			**Diterpenes (1)**				**0.20**		
90	1974	1977	Sclarene				0.20		
			**Others (30)**	**80.73**	**80.07**	**97.51**	**80.72**	**79.15**	**85.03**
1	891	888	Styrene			0.06			
2	931	925	Cumene				0.24		
6	960	960	Benzaldehyde	0.31	0.36	0.48	0.23	0.16	0.15
18	1045	1049	Salicylaldehyde			0.08			
21	1065	1072	Acetophenone	0.01	0.01	0.02			
24	1100	1100	Isopentyl-2-methyl butanoate					0.04	
25	1107	1107	Phenylethyl alcohol			0.27	0.02		
30	1161	1161	Hydrocinnamaldehyde			0.37	0.38	0.46	
35	1196	1195	Estragole				0.14		
37	1219	1218	*Z*-Cinnamaldehyde	0.22	0.25	0.45	0.51	0.49	0.07
38	1227	1233	Hydrocinnamyl alcohol	0.01	0.02	0.05	0.03		
39	1239	1241	Cuminaldehyde				0.02		
40	1242	1245	*o*-Anisaldehyde			0.37			
41	1258	1252	Phenyl ethyl acetate 2			0.03			
42	1250	1260	Chavicol						0.10
**43**	**1270**	**1285**	***E*-Cinnamaldehyde**	**55.26**	**53.04**	**87.04**	**66.25**	**65.82**	**1.31**
45	1287	1293	Safrole	0.03	0.54				0.96
46	1285	1290	*E*-Anethole	0.07	0.03		0.03		
49	1303	1306	*E*-Cinnamyl alcohol				0.17	0.08	0.10
50	1319	1356	α-methyl Cinnamaldehyde	0.01	0.05				
**51**	**1359**	**1361**	**Eugenol**	**21.35**	**21.69**	**0.17**	**1.80**	**6.78**	**75.77**
52	1368	1371	Hydrocinnamyl acetate	0.02	0.06		0.13	0.08	0.08

54	1404	1403	Methyl eugenol	0.01	0.50		0.02		
56	1394	1419	Vanillin	0.04	0.02				
59	1446	1451	*E*-Cinnamyl acetate	1.36	1.65	0.96	8.43	3.25	1.66
70	1523	1527	Eugenol acetate	0.77	0.78				2.13
**72**	**1529**	**1541**	***o*-Methoxy cinnamaldehyde**	**0.03**	**0.03**	**7.13**	**0.52**	**0.30**	
79	1613	1609	Tetradecanal	0.29	0.11		0.02	0.07	
88	1760	1779	Benzyl benzoate	1.06	1.04	0.04	1.75	1.63	3.30
89	1860	1870	Phenethyl benzoate				0.08		

			**Monoterpene hydrocarbons**	**7.48**	**8.36**	**0.23**	**11.07**	**9.95**	**3.26**
			**Oxygenated monoterpenes**	**4.95**	**5.22**	**0.13**	**3.62**	**6.68**	**3.10**
			**Sesquiterpenes**	**4.87**	**4.80**	**0.75**	**3.36**	**3.60**	**5.74**
			**Diterpenes**				**0.20**		
			**Others**	**80.73**	**80.07**	**97.51**	**80.72**	**79.15**	**85.03**
			Total	98.03	98.45	98.62	98.97	99.38	97.13

[a] The number refers to the elution order, and the values (relative peak areas) represent the averages of 3 determinations.
[b] Literature retention index (RI). [c] Retention index (RI) relative to a standard mixture of *n*-alkanes on an SPB-5 column.

Table S2. Sequences of the primers used in this study

Gene annotation	Forward primer 5'-3'	Reverse primer 5'-3'	Tm
SLC40A1	TTGGCCGACTACCTGACCTCTGC	ACACAGACACCGCAAAGTGCC	58
SLC11A1	CGACTGGCTGCACGTCTGGG	GGTGATGAGGACGCCACCCC	60
TfR1	AGCCAACTGCTTTCATTTGTGAGGG	ACGGAAGAAGTCTCCACGAGCAG	58
FTH1	CATGACGACCGCGTCCACCTC	TCAAAGCCACATCATCGCGGTC	58
HMOX1	CCAGGTGACCCGAGACGGCT	AGAGCTGGGCAGGTCCAGGG	59
HAMP1	AAGCTCAAGACCCAGCAGTGGG	AAGTTGTCCCGTCTGTTGTGGG	63
Tf	ACCTGAAGCCTGTGGTGGCA	TGCCTCGAAGCTGGTTCATCTGG	58
DGKK	CAACCTGCATCATCATCGCA	CTGGGCAGCGTTCTTGTATC	59
SLC7A11	AAGCTCATTACAGCTGTGGG	TCTCTTCCTGAAAAGGCGTC	57
GSR	CAACGAGCTTTACCCCGATG	GCAGCATTTCATCACACCCA	59
NOS2	ACAGCACATTCAGATCCCCA	AACACGTTCTTGGCATGCAT	59
OXR1	TCTTCAACTTCTGAGGAGGAGG	CATTCTCTTGAACCAAAGGGTC	57
SOD2	AGGATCCACTGCAAGGAACA	TAGTAAGCGTGCTCCCACAC	59
SOD3	CTGGGTGCAGCTCTCTTTTCA	GAGCAGGCAGGAACACAGTAG	60
Nrf2	TCTGTTGCTCAGGTAGCCCC	TCTGTGGAGAGGATGCTGCTG	61
P53	AGGCCTTGGAACTCAAGGAT	CCCTTTTTGGACTTCAGGTG	60
